# Photocatalytically Enhanced Cationic Dye Removal with Zn-Al Layered Double Hydroxides

**DOI:** 10.1186/s11671-017-2173-y

**Published:** 2017-06-07

**Authors:** G. Starukh

**Affiliations:** Chuiko Institute of Surface Chemistry of National Academy of Sciences of Ukraine, 17 General Naumov Street, Kyiv, 03164 Ukraine

**Keywords:** Layered double hydroxides, Adsorption, photocatalysis, Wastewater treatment, Methylene blue photodestruction

## Abstract

Calcined and organo-modified Zn-Al layered double hydroxides (LDHs) were studied as adsorbents and photocatalysts for removal of cationic dye, as namely methylene blue (MB)*.* Zn-Al LDHs with a cationic ratio of 2:4 were obtained by the coprecipitation method. As-synthesized samples were calcined at different temperatures and the phase transformations were investigated by XRD, TG/DTG, and UV–vis-DR methods. The activity of as-synthesized and calcined Zn-Al LDHs under UV light was attributed to the presence of ZnO phase. The amount of ZnO in LDHs can be regulated by varying of Zn/Al ratio and heating temperature. The impact of Zn/Al ratio on photocatalytic activity of LDHs was observed predominant. The calcined Zn-Al LDHs demonstrated low adsorption of MB. The modification of ZnAl LDHs by sodium dodecyl sulfate was performed using a reconstruction method. The organo/LDH nanohybrids demonstrated high adsorption capacity to MB. The removal of MB from solutions with organo/Zn-Al LDHs was enhanced by using UV light due to MB photodestruction.

## Background

The removal of hazardous organic dyes from wastewater and remediation of contaminants in surface water, groundwater is a major problem in the world. Several traditional methods including adsorption, coagulation, flocculation, ozonation, membrane-filtration, ion-exchange, oxidation, and chemical precipitation are known for the treatment of dye-containing effluents [1, 2]. Adsorption is an inexpensive technique which does not require any special set up. In the recent years, many kinds of adsorbents with the catalytic function were developed and used for removal of nitrate, heavy metals, and organic pollutants from water [3–5].

The use of layered double layered hydroxides (LDHs) as alternative materials for the removal organic dyes from aqueous media has been explored [6–8]. LDHs are known as anionic clays and hydrotalcite-like materials. Their basic structure resembles that of brucite, Mg(OH)_2_, when a fraction *x* of divalent cations is isomorphously replaced by trivalent cations, rendering positively charged layers. The chemical composition of LDHs is expressed by the general formula [M^2+^_1−x_M^3+^_x_(OH)_2_][A^n−^]_x/n_ · zH_2_O, where M ^2+^ may be common; Mg^2+^, Zn^2+^, or Ni^2+^, and M ^3+^ may be common; Al^3+^, Ga^3+^, Fe^3+^, or Mn^3+^. A nonframework charge compensating inorganic or organic anion (CO_3_^2−^, Cl^−^, SO_4_^2−^, RCO_2_^−^) is signed as A^n−^; *x* is normally between 0.2−0.4. LDHs layers gain a positive charge by isomorphous substitution of M^3+^ for M^2+^, which is compensated by interlayer anions and water [9].

The thermal treatment of LDHs leads to the losses of physisorbed and interlayer water molecules, OH^-^ groups of layers, and charge-balancing anions. The layered structure collapses and a mixed metal oxide solid solution is formed. The mixed oxides typically possessed the large specific surface areas, thermal stability, and synergic interactions between the different metal components. Therefore, LDHs calcination products have found numerous applications in various catalytic processes [10–12].

Due to their anionic exchange capacity, LDHs are suitable for anionic dyes intercalation and sorption of but they are not applicable for cationic ones. The modification of LDH’s surface with anionic surfactants allows obtaining the composites that are capable to adsorb different types of organic molecules [13, 14]. Sodium dodecyl sulfate (DS) modified LDHs demonstrated extremely high sorption of cationic dyes such as safranine [15], methylene blue [16], and basic blue [17].

Recently, LDHs have been intensively investigated as promising heterogeneous photocatalysts because of their intrinsic photo-response characteristics, low cost, as well as facile preparation and modification [18]. LDHs as photocatalysts showed large energy-conversion efficiency as a result of the high dispersion of active species in a layered matrix, which facilitates the charge separation. Mixed oxides with semiconducting properties are obtained by calcination of appropriate transition metal-containing LDHs. A wide variety of metal cations, such as Zn^2+^ and Ti^4+^, can be introduced into the layers [19, 20]. Their relative proportions may be varied in a wide range affording the possibility of preparation of semiconducting oxides with tunable properties. Photocatalytic applications of LDHs are an interesting emerging field. Several semiconducting mixed oxides derived from LDH, such as Zn-Al [20], Zn-Ce, ZnFe, Zn-Cr [21], Mg-Zn-Al [22], and Zn-Ga [23], have been studied for the photocatalytic degradation of contaminants.

Besides the undesirable color, breakdown products of dyes also exhibit a mutagenic or carcinogenic effect on human beings and their ingestion can cause severe damage to organisms. Chlorine and hypochlorous acid, generated during degradation, are strong toxic oxidants. They can oxidize organic matters and are simultaneously reduced to chloride [24].As an important basic dye used for printing calico, dyeing cotton and leather, MB could cause various harmful effects such as eye burns, irritation to the gastrointestinal tract and to the skin [25].

The high adsorption capacity of Zn-Al interlayers modified with DS for methylene blue (MB) was demonstrated [26]. The presence of photoactive component in the adsorbent can increase the efficiency of Zn-Al LDH-based materials for removal of cationic dye by using UV irradiation. Thus, the optimal conditions for the preparation of Zn-Al LDHs for the removal of cationic dye, such as MB by adsorption and photodestruction, were determined in the present work.

## Methods

### Synthesis of Zn-Al LDHs

All chemicals were analytical grade and used without further purification. Zn-Al LDHs with carbonate as the interlayer anion, with [Zn]:[Al] = 1:2 were synthesized by co-precipitation method at a constant pH similar to [9]. The first solution containing Na_2_CO_3_ (0.5 M) and NaOH (1.5 M) was obtained. The second solution containing a mixture of metal nitrates of Zn(NO_3_)_2_∙6H_2_O and Al(NO_3_)_3_∙9H_2_O (total metal concentration was 0.6 M, the molar ratio of Zn/Al 2:1, 3:1, 4:1) was prepared and drop wisely added to the first solution under stirring. The pH was adjusted to 10 by addition of NaOH. Once addition was completed, the temperature was raised up to 85 ^o^C and the slurry was being kept for 6 h at this temperature under continuous stirring. After that, the slurry was cooled down to the room temperature within several hours. The product was isolated by filtration and washed with the deionized water until pH 7 several times. Afterward, the solid was dried at 100 °C. The samples were labeled as ZnAl_21_ LDH, ZnAl_31_ LDH, and ZnAl_41_ LDH.

Te above-synthesized Zn-Al LDHs were calcined at 450 °C for 2 h and at 600°C for 1, 2 and 5h in air. The samples were labeled as ZnAl_21_-450, ZnAl_31_-450, ZnAl_41_-450, ZnAl _21_-600-1, ZnAl _31_-600-1, ZnAl _41_-600-1, ZnAl _21_-600-2, ZnAl _31_-600-2, ZnAl _41_-600-2, ZnAl _21_-600-5, ZnAl _31_-600-5, ZnAl _41_-600-5.

The Zn-Al LDHs were modified with sodium dodecyl sulfate CH_3_(CH_2_)_11_SO_4_Na by reconstruction method. The suspensions of 1 g calcined LDHs and 50 ml of 0.05 M DS aqueous solutions were stirred for 24 h at room temperature. The obtained composites were labeled as ZnAl _21_-450/DS, ZnAl _31_-450/DS, ZnAl _41_-450/DS, ZnAl _21_-600-1/DS ZnAl _31_-600-1/DS ZnAl _41_-600-1/DS.

### Characterization

XRD patterns of the samples were recorded with a DRON-4-07 diffractometer (Burevestnik Inc., St. Petersburg, Russia), (CuK_α_ radiation). The thermogravimetric analysis (TGA) and differential thermal analysis (DTA) were carried out using Derivatograph Q-1500 D apparatus (MOM, Hungary) operated in flowing air at a heating rate of 10°min^−1^. Diffuse reflectance spectra were obtained with a Lambda 35 UV-Vis (Perkin Elmer, Germany) spectrometer equipped with an integrating Labsphere RSA—PR-20 in the range of wavelength 200-1000 nm. The UV-visible spectra of the solutions were recorded using a Lambda 35 UV-Vis spectrometer (Perkin Elmer, Germany).

### Photocatalytic Experiment

0.020 g of Zn-Al LDHs was dispersed in 40 mL of 9 × 10^-5^ M (for calcined LDHs) and 10^-4^ M (for DS-modified LDHs) MB aqueous solution in the quartz reactor. Before illumination, the suspensions were stirred for 1 h (calcined LDHs) and 24 h (DS modified LDHs) in the dark in order to reach an adsorption–desorption equilibrium between the photocatalyst and MB molecules. Then, the solution was irradiated for 3 h with mercury vapour lamp (λ_max_ = 365 nm) under magnetic stirring. At given time intervals, the solution was analyzed by measuring the absorption spectra using a UV-Vis spectrometry.

## Results and Discussions

### Characterization

**Fig. 1:**
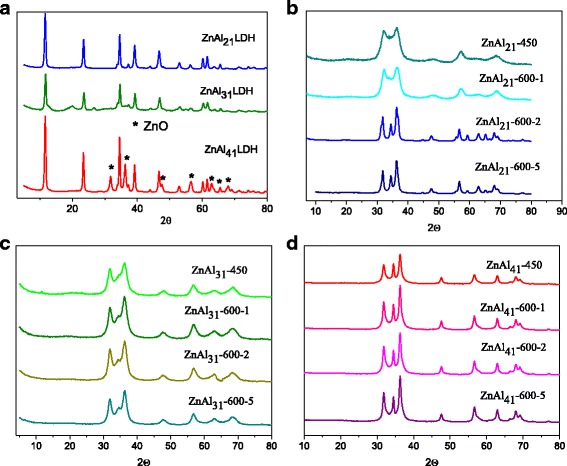
X-ray diffraction patterns of as-synthesized Zn-Al LDHs (**a**) and calcined: ZnAl_21_LDH (**b**), ZnAl_31_LDH (**c**), ZnAl_41_LDH (**d**)

The XRD pattern for the as-synthesized Zn–Al LDHs with the different Zn^2+^/Al^3+^ cationic ratio are presented at Fig. 1a. The hydrotalcite-like structure was formed for all the cationic ratios. The XRD patterns exhibit the characteristic reflections related to the layered double hydroxides. An additional ZnO phase was present in the ZnAl_41_ LDH as indicated by the XRD patterns. The 2θ peaks at 31.9°, 34°, and 36.2° belong to ZnO phase formed on the brucite-like sheets surface. All the reflections are sharp indicating of a highly crystalline material.

The analysis of XRD patterns of the calcined derivatives showed that the layered structure of the original LDHs was completely destroyed suggesting almost total decomposition of the original LDHs and elimination of most interlayer carbonate anions and water (Fig. 1b–d). All reflections could be perfectly indexed as the hexagonal wurtzite structure ZnO. No characteristic reflections corresponding to Al_2_O_3_ phases were observed in the XRD patterns. It must be noted that the higher degree crystallinity of ZnO was improved with increasing of Zn^2+^/Al^3+^ cationic ratio.

**Fig. 2:**
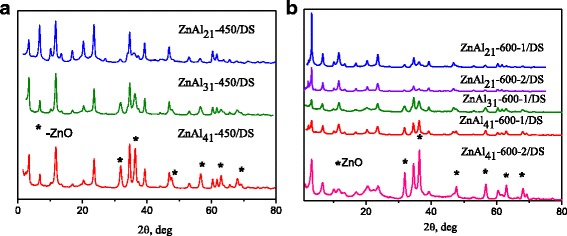
X-ray diffraction patterns of DS modified Zn-Al LDHs obtained by reconstruction of  LDHs calcined at 450 °C (**a**) and 600 °C (**b**)

It is known that the hydration of calcined Zn-Al LDHs in aqueous suspension caused to the reconstruction of the hydrotalcite phase [9]. It could be seen that the layered structure was restored under hydration of calcined Zn-Al LDHs in aqueous solutions of DS (Fig. 2). The appearance of diffraction patterns at small angles evidenced about the presence of DS-intercalated LDHs. All the reconstructed DS modified Zn-Al LDHs contained also carbonate-intercalated phase. It should noteworthy that the complete reconstruction of the layered structure was observed only for ZnAl_21_-450/DS LDH (Fig. 2a). The XRD patterns of ZnAl _31_-450/DS and ZnAl _41_-450/DS LDHs contained ZnO reflections (Fig. 2a). According to [27] the hydration of Zn-Al mixed oxides with Zn/Al ratio 1:5 resulted in the formation of hydrotalcite structure with Zn/Al = 2, irrespective of the initial Zn/Al ratio. So, ZnAl _31_-450/DS, ZnAl _41_-450/DS LDHs contained a lower amount of DS-intercalated phase. The XRD patterns of all DS modified LDHs obtained from Zn-Al mixed oxides calcined at 600 °C contained reflections of ZnO phase (Fig. 2b). Evidently, that the continuous release of Zn ^2+^ from the amorphous oxide phase led to formation of more ZnO nanoparticles with increasing of calcination temperature.

**Fig. 3:**
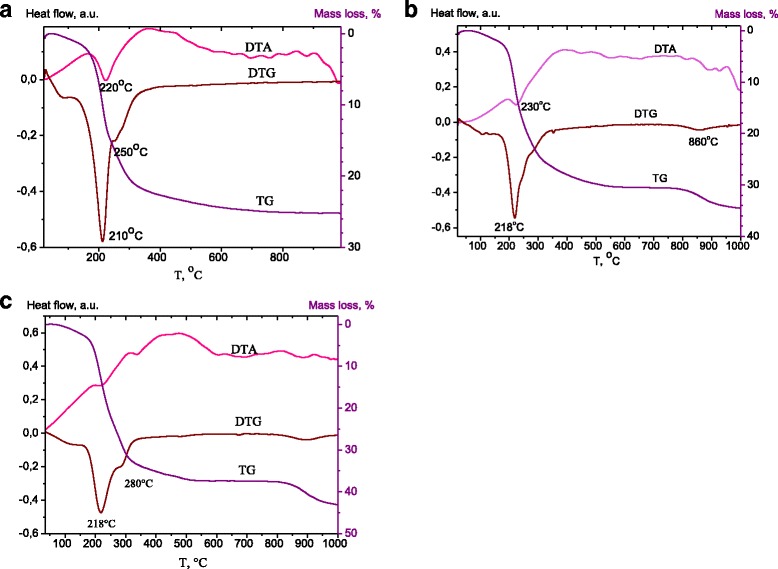
TG, DTA, and DTG curves of ZnAl_41_ LDH (**a**), ZnAl_41_-450/DS (**b**), ZnAl_41_-600-1/DS (**c**)

The thermogravimetric traces recorded for Zn-Al LDHs were very similar for all Zn/Al ratios. TG, DTA and DTG curves for the samples with Zn/Al = 4:1 are presented in Fig. 3. The TGA plot for LDH-carbonate (Fig. 3a) showed the loss of mass in the temperature ranges of 60–190, 190–300, and 300–500 °C. The mass loss in the first step is a common characteristic of hydrotalcite related to the release of physisorbed and interlayer water. The second mass loss was ascribed to the first step of dehydroxylation and the removal of carbonate ions from the interlayer. Over this temperature range, the hydrotalcite underwent decarbonatation and dehydroxylation reactions resulting in the metal oxides formation. In the third step of mass loss that occurred at higher than 500 °C, the mass loss was recognized as the total dehydroxylation, and a collapse of the structure due to the removal of the remaining interlayer anions [28]. The insignificant mass loss observed at 500 – 1000 °C can be ascribed to the loss of some carbonate anions that are strongly adsorbed on the mixed oxides crystallites [29].

The thermal decomposition stages for all studied Zn-Al LDHs are presented in Table 1. The total mass loss decreases with increasing the Zn/Al ratio in ZnAl LDHs, as the materials with lower charge density contain less carbonate anions. Additionally, as ZnAl_31_ LDH and ZnAl_41_ LDH contained the phase of ZnO, the less quantity of metal hydroxides and interlayer carbonate ions was present in the samples. So, the processes of dehydroxylation and decarboxylation for ZnAl_31_ LDH and ZnAl_41_ LDH were not as intensive as for ZnAl_21_ LDH.

**Table 1:** TG and DTG results for the Zn-Al LDHs

Sample	Heat flow step, °C				Mass loss, %
	60–190	190–300	300–500	500–950	
ZnAl_21_ LDH	4.2	20.9	4.4	2.6	32.1
ZnAl_31_ LDH	6.9	19.7	3.3	1.5	31.2
ZnAl_41_ LDH	5.2	15.2	3.2	1.5	25.1
ZnAl_21_ LDH reconstructed	3.9	17.6	4.0	2.0	27.5
ZnAl_21_-450/DS	6.7	28.3	7.6	8.8	51.4
ZnAl_31_-450/DS	4.9	22.0	5.7	5.5	38.1
ZnAl_41_-450/DS	3.7	20.5	5.6	4.3	34.1
ZnAl_21_-600-1/DS	6.6	27.8	7.4	6.2	48.0
ZnAl_31_-600-1/DS	4.6	24.9	9.0	6.3	44.8
ZnAl_41_-600-1/DS	5.0	25.8	6.2	5.3	42.3

The thermogravimetric traces recorded for ZnAl_41_-450/DS presented at Fig. 3b. The first step of the thermal decomposition was attributed to the loss of interlayer water. The second step of decomposition, the dehydroxylation of the brucite-like sheets, was accompanied by DS destruction. The decomposition of DS ions took place in the range of 210–250 °C [30, 31] and, therefore, a greater loss was observed below 200 °C. The mass loss of DS modified LDHs at 300–500 °C was ascribed to the total dehydroxylation and the collapse of the layered structure. The increase in mass loss in this stage resulted from the loading of DS, whose decomposition was reflected by the mass loss in 400–900 °C. The mass loss between 800–900 °C could be recognized as SO_3_ evolution due to the decomposition of (Zn, Al) sulfate formed by decomposition of DS during the second mass loss stage [32].

The total mass losses for ZnAl_31_-450/DS and ZnAl_41_-450/DS were less in comparison with ZnAl_21_-450/DS pointed on the lower content of DS- intercalated phases in the samples with Zn/Al ratio 3:1 and 4:1. The presence of ZnO reflections in the patterns of these LDHs indicated the incomplete reconstruction of LDH under rehydration of mixed double oxides in an aqueous solution of DS (Fig. 2a). According to [33], the extra-phases coexist in the LDHs. The as-synthesized and rehydrated Zn-Al LDHs (Zn:Al = 2:1) contained approximately 25 and 23 wt.% of an amorphous phase [33]. Authors found that the rehydrated samples contained an additional about 3 wt. % of ZnO phase (zincite) resulted from the segregation of Zn from the brucite-like layers. Probably the modification of ZnAl_21_ LDHs with DS caused the additional formation of the amorphous zink hydroxide phase. As suggested in [27], there was a preliminary reaction of rehydration at an early stage of reconstruction of amorphous zinc hydroxide phase and then a rehydration of the Zn-Al oxides during the reconstruction process. Possibly, the rehydration of amorphous phase resulted in the formation of carbonate-intercalated phase. The rehydration of Zn-Al oxides in DS solutions caused to the formation of DS-intercalated phase. The mass losses for ZnAl_21_-600-1/DS, ZnAl_31_-600-1/DS, ZnAl_41_-600-1/DS were isignificantly different. Obviously, Zn-Al LDHs calcined at 600 °C contained less amorphous phase.

**Fig. 4:**
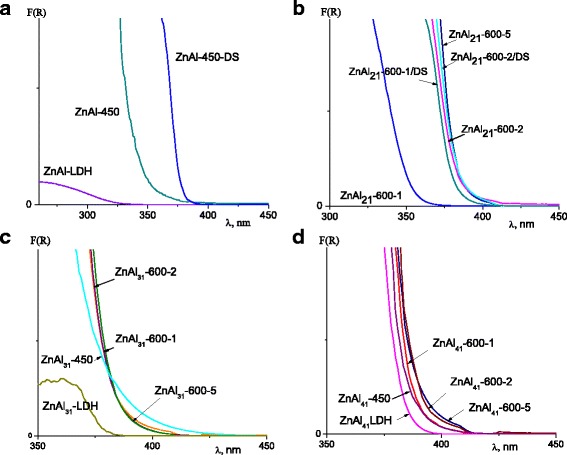
UV-Vis spectra of Zn-Al LDHs with Zn/Al ratio: 2:1 (**a**, **b**), 3:1 (**c**), 4:1 (**d**)

The presence of photoactive ZnO extends the range of applications for LDHs and LDH-based composites, particularly as photocatalysts, UV filters, dye-sensitized solar cells. The effect of phase transformations on Zn-Al LDHs capability to absorb UV light was examined. The optical absorption spectra of as-synthesized Zn-Al LDHs, calcined Zn-Al LDHs and DS modified Zn-Al LDHs for samples with Zn:Al =2:1 are shown in Fig. 4a, b. The reconstruction of mixed oxides calcined at 450 and at 600 °C for 1h promoted the formation of an additional amount of crystalline ZnO resulting in the red shift of absorbance band of ZnAl_21_-450/DS and ZnAl_21_-600-1/DS (Fig. 4a, b). The absorbance band of ZnAl_21_-600-2 was shifted to visible light by approximately 35 nm. There was no change in the absorbance of mixed oxides with Zn:Al = 2:1 obtained by calcinations of LDH for 5 h (Fig. 4b).

The position of absorbance band of mixed oxides with Zn:Al = 3:1 was almost similar for the samples treated at 450 and 600 °C and was independent on thermal treatment duration (Fig. 4c). For Zn-Al LDHs and mixed oxides with Zn:Al = 4:1, the absorption bands were situated at 382–390 nm (Fig. 4d).

Since Al_2_O_3_ is a wide band gap (5.55 eV) material, the absorption of light in the UV range caused by ZnO presented in the samples, the band gap of which is 3.37 eV [34]. The values of band gap energy (E_g_) of the samples were calculated from the intercept of UV–vis spectra using the equation: E_g_ = 1240/λ [35] (Table 3). Among calcined Zn-Al LDHs the lowest value of band gap energy was observed for the sample with the highest content of Zn (Table 3).

### Photocatalytic Study

In order to evaluate the photocatalytic performance of Zn-Al LDHs, the degradation of aqueous 2*10^-5^ M MB solution under UV light was conducted. The poor light absorption ability of LDHs with Zn/Al ratio 2:1 and 3:1 caused its low activity under irradiation (Table 2).

**Table 2:** The photocatalytical MB removal with as-synthesized and calcined Zn-Al LDHs

Sample	Bandgap, eV	Adsorption		Photodestruction		Total removal, %
		%	mg/g	%	mg/g	
Blank	–	–	–	25		25
ZnAl_21_ LDH	3.81	6	0.8	26	3.1	32
ZnAl_31_ LDH	3.30	7	0.9	11	1.3	18
ZnAl_41_ LDH	3.31	5	0.6	72	9.2	77
ZnAl _21_-450	3.78	3	0.4	51	6.5	54
ZnAl_21_-600-1	3.60	4	0.4	68	8.7	72
ZnAl_21_-600-2	3.30	6	0.8	74	9.4	80
ZnAl_21_-600-5	3.32	6	0.8	78	10.0	84
ZnAl _31_-450	3.30	7	0.9	51	6.5	58
ZnAl_31_-600-1	3.32	6	0.8	70	8.4	76
ZnAl_31_-600-2	3.31	6	0.8	76	9.1	82
ZnAl_31_-600-5	3.31	6	0.8	80	9.6	86
ZnAl _41_-450	3.29	4	0.4	70	8.5	74
ZnAl_41_-600-1	3.24	5	0.6	90	10.8	95
ZnAl_41_-600-2	3.22	4	0.4	95	11.4	99
ZnAl_41_-600-5	3.22	6	0.8	92	11.0	98

**Fig. 5:**
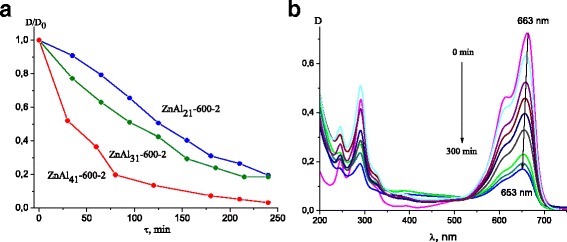
Degradation curves of MB over calcined Zn-Al LDHs under UV-light irradiation (**a**); Absorption changes of 2*10^-5^ M MB solution during the photodegradation process over ZnAl_31_-600-1 (**b**)

ZnO-contained ZnAl_41_ LDHs among the other LDHs demonstrated the higher photocatalytic activity in the destruction of MB. The photocatalytic activity was highly improved when samples were calcined at 450 and 600 °C due to the larger amounts of ZnO phase formed. The MB photodegradation curves for Zn-Al LDHs calcined at 600 °C are presented in Fig. 5a. Since the difference in photocatalytic performance of calcined Zn-Al LDHs was caused by ZnO phase, the LDHs with Zn/Al ratio 4:1 demonstrated the best results in MB photodestruction.

The changes in the absorbance spectra of MB solution over different time of irradiation in the presence of ZnAl_31_-600-1 can be seen in Fig. 5b. The peaks at 610 and 663 nm were assigned to the absorption of the conjugated π-system, while the peaks close to 300 nm were assigned to the absorption of the aromatic ring [36]. It could be seen that the intensity of original peaks was decreased with the increase of irradiation time. Besides, a parallel decrease in intensities and slight blue shift of the bands located at 663 nm could also be observed. It was caused by the N-demethylation of the phenothiazine and its concomitant degradation [37]. The similar changes in optical absorbance spectra of MB were observed for photocatalytic systems with all LDHs.

DS modification of Zn-Al LDHs increased its affinity for MB due to hydrophobic interactions between the surfactants and dye molecules [26]. ZnAl_41_-600-1/DS demonstrated the best result on MB adsorption (Table 3). The photocatalytic activity of organo/Zn-Al LDHs was comparable with calcined Zn-Al LDHs (Table 3). The higher level of MB degradation was observed for the ZnAl_41_-450/DS. The values of removal of MB with DS modified Zn-Al LDHs by adsorption were higher than that of by photodestruction*.* So, the adsorption removal of MB with organo/Zn-Al LDHs could be enhanced by applying of light irradiation.

**Table 3:** The adsorption and photocatalytic removal of MB with DS modified Zn-Al LDHs

Sample	C_0_, mol/l	Adsorption		Photocatalysis		Total removal	
		%	mg/g	%	mg/g	%	mg/g
ZnAl_21_-450/DS	0.9*10^-4^	85	49	7	4	92	53
ZnAl_31_-450/DS	0.9*10^-4^	76	44	17	10	93	54
ZnAl_41_-450/DS	0.9*10^-4^	75	43	21	12	96	55
ZnAl_21_-600-1/DS	0.9*10^-4^	73	42	15	9	88	51
ZnAl_31_-600-1/DS	1.0*10^-4^	78	48	16	10	94	58
ZnAl_41_-600-1/DS	1.0*10^-4^	93	58	4	2	97	60
ZnAl_41_-600-2/DS	1.0*10^-4^	69	43	10	6	79	49

Probably, the photodestruction of dye has been occurred on the surface of DS modified Zn-Al LDHs that was not fixed under the experimental conditions. After equilibrium achievement, the DS modified Zn-Al LDHs with adsorbed MB had intensive blue color.

## Conclusions

In this work, the as-synthesized and calcined Zn–Al LDHs with different cationic ratios were prepared. The obtained materials were characterized and used for cationic dye MB removal from aqueous solutions. The studies of MB photodestruction under UV light over both LDHs and calcined LDHs indicate that:

The photocatalytic activity of Zn–Al LDHs was originated from the presence of ZnO phase. The formation of ZnO phase in LDHs could be regulated by the increase of Zn/Al ratio in LDHs and with the temperature treatment of LDHs.

The influence of Zn/Al ratio on photocatalytic activity of LDHs was predominant. The photodegradation of MB in the present of the as-synthesized ZnAl_41_ LDH and ZnAl_41_ LDH at 600 °C was 72 and 95%, respectively. For calcined at 600 °C ZnAl_31_ LDH and ZnAl_21_ LDH, the photodegradation of MB were 76 and 74%, respectively.

The organo/Zn-Al LDHs showed high adsorption capacity to cationic dye MB. They also demonstrated the photocatalytic activities in the MB destruction. Hence, the adsorption removal of MB from solutions with DS modified Zn-Al LDHs could be enhanced by using UV irradiation.
